# Digital Semiology: A Prototype for Standardized, Computer-Based Semiologic Encoding of Seizures

**DOI:** 10.3389/fneur.2021.711378

**Published:** 2021-10-05

**Authors:** Tal Benoliel, Tal Gilboa, Paz Har-Shai Yahav, Revital Zelker, Bilha Kreigsberg, Evgeny Tsizin, Oshrit Arviv, Dana Ekstein, Mordekhay Medvedovsky

**Affiliations:** ^1^Department of Neurology, Agnes Ginges Center for Human Neurogenetics, Hadassah Medical Organization, Jerusalem, Israel; ^2^Faculty of Medicine, The Hebrew University of Jerusalem, Jerusalem, Israel; ^3^Pediatric Neurology Unit, Hadassah Medical Organization, Jerusalem, Israel; ^4^The Leslie and Susan Gonda (Goldschmied) Multidisciplinary Brain Research Center, Bar-Ilan University, Ramat Gan, Israel; ^5^School of Nursing, The Hebrew University of Jerusalem, Israel and Hadassah Medical Organization, Jerusalem, Israel

**Keywords:** epilepsy, video-EEG monitoring, seizure classification, epilepsy surgery, SUDEP, PNES

## Abstract

Video-EEG monitoring (VEM) is imperative in seizure classification and presurgical assessment of epilepsy patients. Analysis of VEM is currently performed in most institutions using a freeform report, a time-consuming process resulting in a non-standardized report, limiting the use of this essential diagnostic tool. Herein we present a pilot feasibility study of our experience with “Digital Semiology” (DS), a novel seizure encoding software. It allows semiautomated annotation of the videos of suspected events from a predetermined, hierarchal set of options, with highly detailed semiologic descriptions, somatic localization, and timing. In addition, the software's semiologic extrapolation functions identify characteristics of focal seizures and PNES, sequences compatible with a Jacksonian march, and risk factors for SUDEP. Sixty episodes from a mixed adult and pediatric cohort from one level 4 epilepsy center VEM archives were analyzed using DS and the reports were compared with the standard freeform ones, written by the same epileptologists. The behavioral characteristics appearing in the DS and freeform reports overlapped by 78–80%. Encoding of one episode using DS required an average of 18 min 13 s (standard deviation: 14 min and 16 s). The focality function identified 19 out of 43 focal episodes, with a sensitivity of 45.45% (CI 30.39–61.15%) and specificity of 87.50% (CI 61.65–98.45%). The PNES function identified 6 of 12 PNES episodes, with a sensitivity of 50% (95% CI 21.09–78.91%) and specificity of 97.2 (95% CI 88.93–99.95%). Eleven events of GTCS triggered the SUDEP risk alert. Overall, these results show that video recordings of suspected seizures can be encoded using the DS software in a precise manner, offering the added benefit of semiologic alerts. The present study represents an important step toward the formation of an annotated video archive, to be used for machine learning purposes. This will further the goal of automated VEM analysis, ultimately contributing to wider utilization of VEM and therefore to the reduction of the treatment gap in epilepsy.

## Introduction

Epileptic seizure assessment using video-EEG monitoring (VEM) is paramount in the workup of patients with drug resistant epilepsy and in many patients with suspected epilepsy. Despite technological advances in EEG analysis and source localization, seizure semiology remains fundamental in localization of the symptomatogenic zone in candidates for non-pharmacological treatments. It is also pertinent in differentiating between epileptic and non-epileptic etiologies or identifying specific epileptic syndromes.

VEM interpretation is still performed in most institutions using a freeform report, without a standardized approach to the semiologic description of seizures, therefore posing several challenges. Firstly, the semiologic analysis of seizures is a time-consuming task, performed by highly trained experienced personnel, limiting the availability of this important diagnostic tool, and raising its cost. Also, certain semiologic aspects of the seizure may be overlooked, increasing the inter-rater variability (1). In addition, when compared to a freeform report, standardized VEM reports hold the added value of enabling implementation of machine learning algorithms on the data.

Of previous initiatives aimed to standardize the EEG report, most noteworthy is that of the SCORE taskforce (2), which focuses on EEG descriptors but also addresses seizure semiology. In the SCORE algorithm over 100 semiologic features are divided into early and late ictal, as well as postictal timeframes, encoded in sequential order but without specifying the exact timing of each semiologic feature in the seizure. ASTEP, another seizure semiology annotation software, allows for better chronologic accuracy but contains less semiologic descriptors (3).

To address the above-mentioned issues, we designed a novel systematic, computer-based approach to semiologic annotation of seizures. We created a video annotation software, “Digital Semiology” (DS), consisting of predefined hierarchal semiologic descriptors that can be selected to describe seizure semiology. The software allows for a temporal resolution of 1 s and contains over a hundred semiologic descriptors to maximize report precision, while maintaining feasibility of use by its hierarchical structure. In addition to standardization of the VEM report, the DS software contains a semiologic extrapolation function, which automatically identifies key semiologic aspects in the completed report that may be compatible with a focal episode, a PNES (psychogenic non-epileptic seizure), Jacksonian march and increased risk of SUDEP (sudden unexpected death in epilepsy patients), as a proof of concept for the development of future comprehensive automatic interpretation.

The purpose of the current pilot study is to present the DS software, demonstrate its feasibility of use and compare its precision with a freeform report, as well as present preliminary implementation of the semiologic extrapolation functions.

## Methods

### DS Software

**Figure 1:**
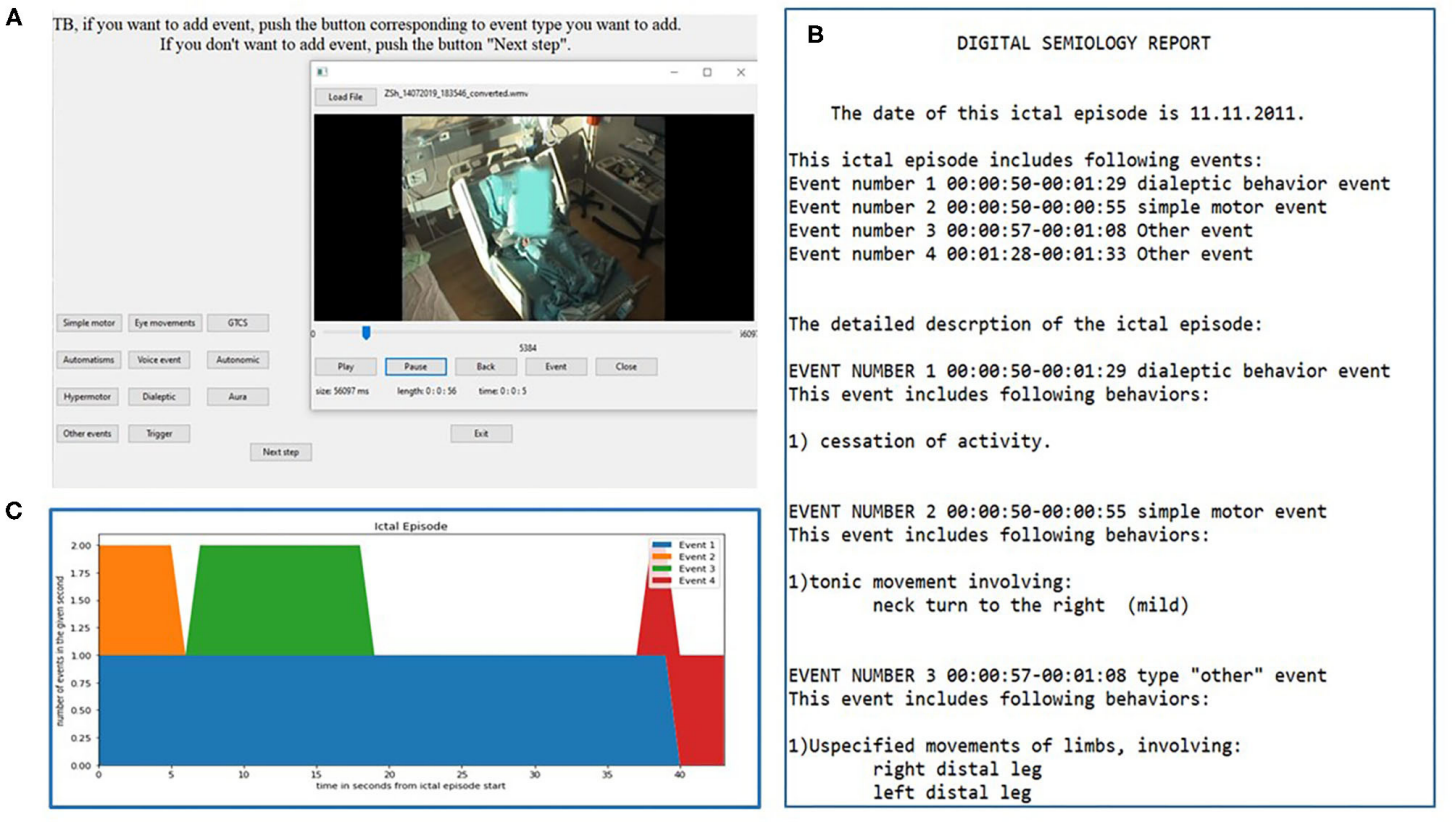
Digital semiology interface and report. The interactive window for video annotation is shown in **(A)**. The encoder can move back and forth within the video and the timing selected is automatically inserted into the report. The example shows the “event type” selection window. An example of the final report and its graphic representation are seen in **(B,C)**, respectively.

The DS software is a python programmed console freely available for download at https://sites.google.com/view/digital-semiology/. The software enables the user to view video excerpts and annotate their semiologic characteristics (Figure 1A). The software automatically generates a written report (Figure 1B) as well as a graphic scheme of the different events (Figure 1C).

The semiologic descriptors used in DS are based largely on known semiologic classification schemes (4, 5). However, since the program is meant to facilitate the encoder in describing viewed behaviors, certain adaptations have been made. In addition, DS allows users to write free-text comments, which are integrated into the report.

#### Hierarchal Structure of DS

Each episode encoded using the DS software is divided into three hierarchal categories: (1) the ictal episode, (2) ictal events, and (3) ictal behaviors. The ictal episode is the highest category and provides the timeframe for the entire encoded episode. For each episode the user is asked to define whether the ictal episode was likely representative of baseline behavior, a PNES, a non-epileptic and non-psychogenic event (e.g., syncope), and whether it seems to have started from sleep. These questions are meant to structuralize the user's general impression of the episode, which represents either an epileptic seizure or other types of fits. After completion of the encoding, the user is also required to note any behaviors that may have seemed post ictal rather than ictal.

Within the ictal episode, the user may define ictal events belonging to one of eleven categories (Table 1) and determine the timeframe for each event. The timeframes of the events are allowed to overlap. Within each event, the user may define one or more ictal behaviors belonging to the event category, which are contained within the predetermined event timeframe.

**Table 1:** Event types in DS, and the behavioral categories appearing next in the DS hierarchy.

**Event type**	**Behavioral categories**
Simple motor	Tonic, dystonic, clonic, myoclonic, fencer posturing, myoclonic, negative myoclonus, atonic, atactic, apractic, tremor, paralysis, epileptic spasms, figure-of-4.
Automatisms	Limb gesticulating/repetitive movements, limb semi-purposeful movements, limb raising, nose wiping, ear plugging, genital manipulations, hand-mouthing, stepping, pedaling, oral automatisms, yawning, facial expressions, spitting, vomiting, cough, sneezing, pelvic thrust, urinary behavior, defecation, other.
Autonomic	Hyperventilation, hypoventilation, apnea, dyspnea, stridor, piloerection, pallor, flushing, cyanosis, sweating, mydriasis, miosis, Cheyne Stokes breathing, irregular breathes, hiccups, salivation, urine loss, fecal loss, other.
Eye movements	Gaze deviation, nystagmus, chaotic eye movements, blinking, eyelid myoclonus, blepharospasm, eyelid retraction, eyelid flutter with closed eyes, eyes open, eyes closed.
Hyperkinetic	Bimanual bipedal automatisms, gyratory movement, rocking.
Voice phenomena	Non-verbal vocalization without panic behavior, non-verbal vocalization with panic behavior, coherent speech, panic speech, cursing, confused speech using comprehensible words, perseverations, paraphasia, motor aphasia, sensory aphasia, sensorimotor aphasia, naming difficulty, laughter, crying, stuttering, unintelligible language, snoring, dysarthria, hoarseness, other.
Dialeptic	Completely irresponsive, partially irresponsive, amnesia, cessation of activity, slowing of activity, general muscle hypotonia, exploratory behavior, non-verbal aggressive behavior, non-verbal panic behavior, agitation, disoriented behavior, other.
GTCS	
Aura	Somatosensory, visual, auditory, gustatory, olfactory, vertiginous, autonomic/visceral, experiential, cephalic, other.
Other	Vigilance change, patient's position change, walking, observer's behavior change, patient moves out of the video-camera field, head nodding, side-to-side head movements, unspecified head movements, unspecified movements of trunk/face/limbs, falling, eating, drinking, physical trauma, periodic limb movements of sleep, chorea, athetosis, akathisia, tics, balismus, unspecified dyskinesia, unspecified dystonia, body part shaking, pelvic thrust, other.
Trigger	Visual, music, auditory non-musical, tactile, startle, eating, drinking, other.

*Many of these categories lead to additional descriptors in the DS hierarchy, such as force of movement, involved body parts etc. (see full DS hierarchal scheme in Supplementary Materials)*.

The unique hierarchal structure of DS accommodates a wide variety of semiologic descriptors but displays only a limited and relevant selection at any given moment, depending on prior user selections. Thus, events precede behaviors in the hierarchy, which, in turn, may also be constructed in a hierarchal fashion. Thus, the user may select relevant involved body parts or specify particular behavioral subtypes if these are relevant to the selected behavior. For example, when selecting a tonic behavior within a simple motor event, the user can specify the involved body parts. If the distal upper limb is involved, specific hand positions, such as politician's fist, cup, extended hand, pointing, pincer, and fist, can be selected (see Supplementary Material for the entire DS scheme).

#### Semiologic Extrapolation in DS

The semiologic extrapolation function in the DS software implements a set of predetermined rules on the final report. Its aim is to identify specific semiologic elements which can be associated with a variety of event types, and at present it contains four functions, which identify features implying focal onset, PNES, the presence of Jacksonian march and semiologic risk factors for SUDEP. The final report includes an alert detailing specific semiologic features compatible with each of the enabled semiologic extrapolation functions. The semiologic extrapolation functions generate either a possibly positive statement or inconclusive (“don't know”) statement. They are designed as decision support functions, highlighting key semiologic elements, and always require clinical judgment and EEG considerations to assist diagnostic process. For example, if the video report includes all criteria for PNES and DS raises the possibility of PNES, but the ictal EEG is clearly epileptiform, the interpreter should ignore the DS suggestion and define the episode as an epileptic seizure.

The focality feature checks for the presence of semiologic characteristics of focal, rather than generalized seizure onset (Table 2.a). While there are many such characteristics, herein we chose semiologic descriptors which are highly specific. Thus, when observing motor semiologic features, only forced (rather than mild or non-forced) head and eye deviation are reported by the focality function (6). In addition, unilateral movement, whether tonic, clonic or dystonic, must appear at least 5 s before contralateral movement so that equivocal cases, in which it may be uncertain that the movement truly commenced unilaterally, are not falsely classified as focal. Similarly, unilateral hemiparesis must appear at least 5 s before contralateral involvement. Fencer and figure of four posturing which are highly lateralizing were also included. In its current form the focality function does not have localizing properties but alerts the user if features suggestive of focal rather than generalized onset were reported in the encoded episode.

**Table 2:** Criteria implemented by the semiologic extrapolation functions in DS.

a. Focality criteria 1. Figure of 4. 2. Fencer posturing. 3. Forced head turn. 4. Forced gaze deviation. 5. Hyperkinetic event at least 5 s before start of GTCS. 6. Aura reporting event at least 5 s before any other event, except of another aura reporting event or trigger event. 7. Unilateral tonic, clonic or dystonic movement of arm or leg or side of face or hemibody at least 5 s before start of contralateral involvement. 8. Unilateral paralysis of arm or leg or side of face or hemibody at least 5 s before start of contralateral paralysis.	b. PNES criteria 1. Patient's eyes were closed during the ictal episode. 2. The patient resisted an attempt to open eyes by observer. 3. The patient completely controlled her/his fall during ictal episode. 4. The duration of ictal episode was longer than 5 min.	c. Jacksonian March 1. Jacksonian march is defined as spatio-temporal clonic movement.propagation on the same body side between face, arm and leg and/or different parts of distal extremities. 2. If between two epochs with clonic movements is an epoch without clonic movements, such sequences are not considered as Jacksonian march. 3. If at the same moment leg and face are simultaneously involved in clonic movements, further propagation is not considered as Jacksonian march. 4. Synchronous bilateral clonic movements (with the same start and end timings) are excluded. However, if not completely synchronous, clonic movements involving both body sides are not excluded, rather considered as to separate sequences.	d. SUDEP 1. Presence of B/GTCS. 2. GTCS started from sleep. 3. GTCS ended with apnea. 4. GTCS ended with cyanosis. 5. GTCS ended in prone position.

*PNES, psychogenic non-epileptic seizures; SUDEP, sudden unexpected death in epilepsy; B/GTCS, bilateral/generalized tonic clonic seizures*.

The PNES feature checks for characteristics shown to be typical of PNES (Table 2.b). Again, the features selected were such that are considered relatively specific in the literature. Eye closure during the ictal episode has been shown to be highly sensitive and specific for PNES (96.2 and 98.1% respectively) (7, 8). PNES are generally significantly longer then epileptic events (9), and a seizure length of over 5 min makes an event 24 times more likely to be PNES (10). Fully controlled falling and resistance to eye opening are also suggestive of PNES (11). Other semiologic characteristics such as head shaking or pelvic thrust were ultimately omitted from the PNES criteria due to low specificity, particularly when compared with frontal lobe epilepsy (11).

The Jacksonian march function (Table 2.c) identifies propagation of clonic body movements between body parts separated by at least 1 s intervals (the temporal resolution of DS).

The SUDEP risk function (Table 2.d) alerts in case of semiologic characteristics which have been shown to increase the risk of SUDEP. These include, first and foremost, the appearance of GTCS (12). GTCS out of sleep, apnea or cyanosis at the end of the GTCS (13) and GTCS ending in prone position (14) are noted as additional risk factors, though the evidence for their contribution to SUDEP risk is less robust.

### Patient and Event Selection

Sixty video excerpts of episodes from 45 adult and 15 pediatric patients were selected from the Hadassah Medical Center VEM archive. Episodes were selected in backwards chronological order starting 6 months prior to the time of beginning DS-guided review, and only discrete events for which a detailed event-specific VEM report was previously written were included. These freeform reports were composed as freetext paragraphs or lists describing the temporal sequence of semiologic events which occurred during the seizure, elaborating selected timepoints at the interpreters' discretion. VEM records were edited according to the EEG and the available freeform report to create video excerpts of episodes devoid of concurrent EEG data. Each one of four epileptologists (TB, TG, MM, and DE), used DS to analyze 15 video excerpts for which they had previously composed a detailed report. This was done to overcome inter-rater variability, allowing intra-rater comparison of the DS and freeform report. At least 6 months had passed between composition of the freeform and DS report, and the epileptologists were blinded to their previous reports.

The review of charts and videos of patients in this study was approved by the Hadassah Medical Organization Institutional Review Board HMO 0548-19. Due to the retrospective nature of the study, the need for informed consent was waived.

### Measures Used to Compare DS and Freeform Report

Quantitative comparison between the freeform and the DS report was performed by enumerating the timepoints and behaviors used in each report. The number of timepoints and behaviors were compared using a paired two-tailed *t*-test. In addition, the content of the reports was compared. Each episode was scored twice: once with an omissions score, namely the fraction of descriptors that appeared in both the DS report and freeform report compared to the freeform report alone; and once with an additions score consisting of the fraction of descriptors that appeared in both the DS report and freeform report compared to the DS report alone. These scores were meant to assess the similarity between the two reports, and underscore both shortcomings (omissions) and advantages (additions) in DS compared to a freeform report.

The duration of coding per episode was calculated based on the start and end time of encoding found in the episode logfile.

## Results

### Event Subtypes in the Seizure Cohort

**Figure 2:**
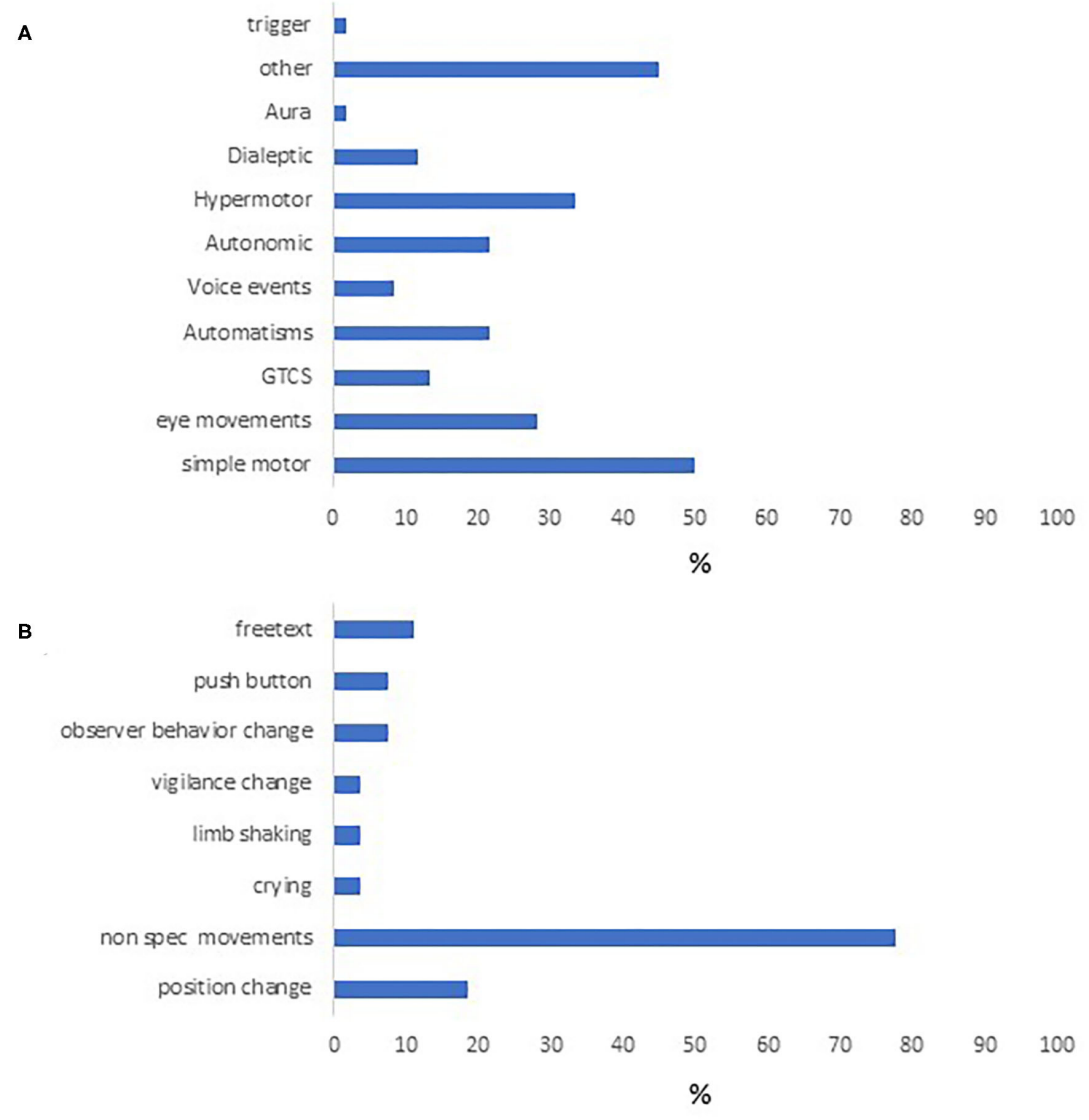
Semiologic characteristics of the study cohort. Event types appearing in our cohort are seen in **(A)**. **(B)** contains the different behaviors under “other,” the most common of which were nonspecific limb movements, followed by position change.

Of the 60 episodes encoded using DS, the most frequent event type was “simple motor”. Only 7 events used the dialeptic module (Figure 2A). “Other” events were also quite common (Figure 2B), and in 21 seizures this was used to depict non-specific movements which did not fall into other movement categories. The “other” module was also used by encoders to describe position changes, button pushes, crying and for free text descriptions.

### Comparison of Temporal and Semiologic Resolution Between DS and the Freeform Report

**Figure 3:**
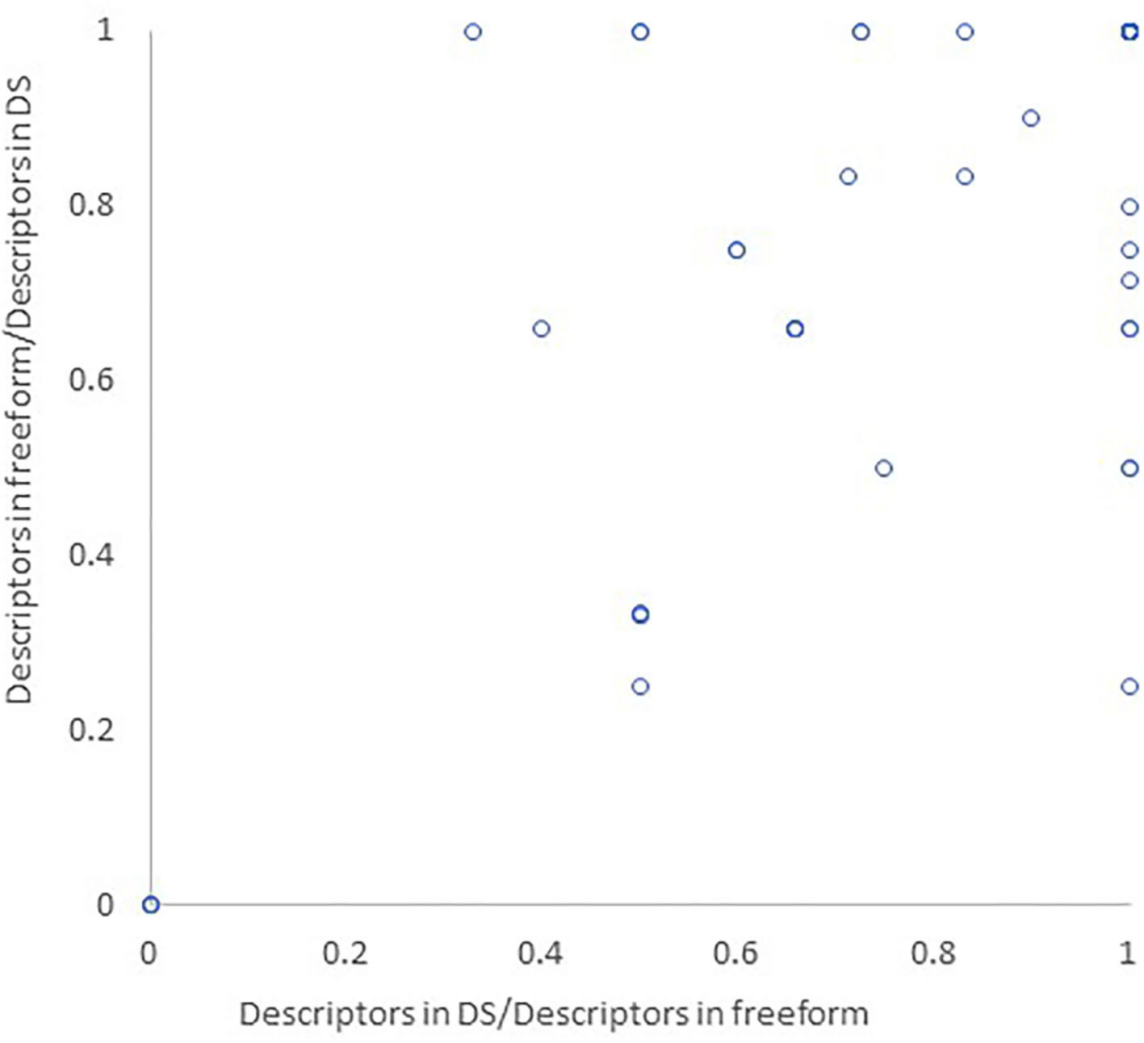
Similarity between DS and freeform reports. The semiologic descriptors appearing in both DS and freeform report were divided by all semiologic descriptors in the freeform report (x axis) and DS report (y axis), corresponding to behaviors omitted from the DS report or added to it, respectively.

The average fraction of descriptors appearing in both the DS and freeform report compared to the freeform report alone was 0.80 ± 0.29. In 36 of the 60 episodes encoded, all semiologic descriptors appearing in the freeform report also appeared in the DS report. The average fraction of descriptors appearing in both the DS and freeform report compared to the DS report alone was 0.780 ± 0.3 (Figure 3).

When examining the specific semiologic descriptors which were omitted in the DS reports, non-specific movements were most commonly omitted, but in one case figure of four posturing and in two cases head version were missing from the report. Other descriptors which were omitted included eye closure or eye opening, breathing patterns, and irresponsiveness.

When comparing the number of timepoints which appeared in each report, the DS report and the freeform report did not differ significantly (3.883 ± 3.02 and 3.915 ± 5.44, respectively, *p* = 0.95). The average number ofs behaviors used to depict each episode was also similar between the DS and freeform report (4.173 ± 0.26 and 5.034 ± 0.77, respectively, *p* = 0.12).

### Time to Code Event

The average episode duration was 1 min and 45 ± 288 s and ranged between 2 and 33 min. The average time to encode an episode using DS was 18 min and 13 s (standard deviation: 14 min and 16 s), with a median of 15 min, ranging between 5 min and 1 h and 40 min.

### Semiologic Extrapolation Function

#### Focality

The semiologic extrapolation function identified 21 episodes as focal, of these 19 were indeed focal based on the concurrent EEG (which was unavailable to encoders using DS), with the remaining two being episodes of PNES. Of the correctly identified focal episodes, forced head turn was seen in 38%, as were unilateral motor manifestations. Hyperkinetic motor manifestations were seen in 23%. Fencer and figure of four posturing were rarer, and seen in 2 and 4 cases, respectively. Twenty-four additional episodes of focal origin were not identified by the focality function. Of these, 10 were dialeptic episodes which were accompanied by automatisms or non-specific movements in 4 cases. Two episodes began with either motor signs or alarm button push and culminated in bilateral tonic clonic seizures. The remaining 12 events consisted of various limb movements, at times described as automatisms, as well as crying (1 episode) and gaze disturbances (1 episode). Overall, the calculated sensitivity of the focality function was 45.45% (CI 30.39–61.15%) and its specificity was 87.50% (CI 61.65–98.45%).

#### PNES

The PNES alert identified 6 out of 12 episodes of PNES in the study cohort, all of which were of long duration and two in which the patient's eyes were closed during most of the event. An additional event identified by the PNES alert was in fact a lengthy focal seizure and that episode was also identified by the focality function. Six additional PNES episodes were not identified by the PNES function. All these episodes consisted of relatively brief events with non-specific movements of the head, shoulders, and limbs. Two of these events were identified by the focality function, one due to the appearance of hyperkinetic movements, and the other due to unilateral dystonic posturing. Overall, the calculated sensitivity of the PNES function was 50% (95% CI 21.09–78.91%) and its specificity was 97.2 (95% CI 88.93–99.95%).

#### Jacksonian March

The Jacksonian march function did not identify any suspicious events in our episode cohort. Based on the freeform reports there were no such events in the cohort.

#### SUDEP Risk Factors Function

The software identified 11 events of GTCS in the cohort and a SUDEP risk alert was included in the report. In one case apnea was noted following the GTCS and in another the event was suspected to have started from sleep, and this additional information was also available in the final report.

## Discussion

This study presents DS, a novel software developed to facilitate VEM interpretation and decoding, with the goal of forming a standardized annotated video archive of seizures, which may be used in machine learning. We describe our first experience using the DS software, showing that it can be relatively easily implemented in a cohort of both adult and pediatric patients, and that the resulting report is similar to a freeform report in terms of temporal resolution and wealth of behaviors described.

### The DS Report as an Alternative to the Freeform Report

The freeform and DS reports were similar in the majority of cases, but mismatches did occur, both due to omissions and additions of semiologic descriptors to DS report. The omissions can be divided into two subgroups: omission of a significant semiologic descriptor such as gaze or head deviation, versus omission of non-specific movements (for example: leg movements under blanket). While the first can hold an important semiologic clue that should not be missed, the second is of uncertain value and its omission perhaps allows the DS report to be more precise. Either way, since the freeform and DS report were encoded by the same physician for each episode, the instances of mismatch between the two highlight, in our eyes, the sometimes-evasive nature of semiologic analysis and the need for standardization of the report.

One cause for mismatch between the DS report and the freeform report lies in the different ways of describing a specific semiologic event. For example, head version can be described as forced or non-forced or even as non-specific head movements by different observers. While the subject of inter-rater variability in description of specific semiologic characteristics has not been systematically studied to the best of our knowledge, there is known variability in describing EEG (15) and it has been assumed that these differences may apply to semiologic characteristics as well (4). In the future, implementing DS as part of a multicenter study in which each event is observed by more than one neurophysiologist may allow to test the question of interrater variability in semiologic analysis of seizures, as well as the applicability of DS beyond our center.

Another explanation for discrepancies between the two reports may stem for the fact that one was construed while viewing the EEG while in the other the interpreter did not have direct access to EEG findings. Since the interpretation of certain semiologic characteristics is subjective (perhaps most notably—behavioral arrest), the absence of the EEG correlate may make the interpretation more challenging. In addition, a VEM report is often composed after viewing several episodes of the same patient, which aids in choosing the most appropriate semiologic descriptors for each behavior and differentiating between seizure-related and random behaviors, but in the study only one episode was available per patient. Adding concurrent EEG findings to future versions of DS will also allow for an automated report which adheres to the 2017 ILAE seizure classification (5).

### The Semiologic Extrapolation Function

The question whether a video event can be interpreted in an informative manner without concurrent EEG was addressed using the semiologic extrapolation function. The criteria formed for the focality alert were strict criteria which are highly suggestive of a focal epileptic event (4). Thus, many criteria, which may be more equivocal, were purposely omitted from the function, which was created with high specificity in mind. Indeed, based solely on seizure semiology, approximately half of focal seizures were identified, with a specificity nearing 90%. Recently, a novel semiologic visualization tool used a rigorously selected semiology database to extrapolate a likelihood score of involvement of various brain regions given a specific semiologic depictor (16). In the future, we hope to create a similar focality score formed of weighted parameters which may improve the sensitivity of the focality function, and aid not only in determining focality, but also in scoring probable involved regions. Additionally, integrated with EEG data as part of an AI-based VEM interpretation system, this function will aid to the localization of frequently equivocal electrographic characteristics of the recordings, similarly to the way such localization is currently obtained in clinical practice.

The PNES function showed similar specificity and sensitivity to the focality function. Interestingly, all identified PNES events were of long duration. The six events missed by the function were briefer motor events. While the availability of EEG aids significantly in differentiating between epileptic events and PNES, some cases may remain equivocal (17). Additionally, differentiating between motor movements which are more typical of PNES as opposed to epileptic seizures may be difficult based exclusively on a written report and is highly encoder dependent. In the future, elaborating the semiologic characteristics associated with the function may aid in improving its sensitivity. In fact, after this study was finalized, we updated the PNES function so that vocalizations lasting 10 s or longer (excluding ictal cry or ictal laughter) were added as a PNES criterion. In addition, three features known to decrease the probability of PNES were included in the function, namely nighttime occurrence, ictal self-injury and urinary incontinence (18). In its current version, the PNES function may generate two statements, one alluding to features which increase the probability of PNES, and the second alluding to features rendering it less likely. Also, better delineating epileptic motor behaviors and sequences may aid in identifying sequences which are likely epileptic (19). Furthermore, other video-related parameters, such as motion speed and frequency may help differentiate between epileptic and non-epileptic events (20). Finally, an integrative PNES score may be useful when considering equivocal VEM cases and perhaps in the future, as the scores are refined and validated, also when the only available information is a video excerpt.

None of the episodes included in the cohort contained a Jacksonian march, and indeed, no Jacksonian march was identified by DS. Since the Jacksonian march rarely occurs, this finding is not surprising. Of note, the Jacksonian march function differs from the focality and PNES functions in that it identifies a motor sequence/ pattern as a semiologic feature. Indeed, defining other specific sequences of motor or semiologic features may aid in maximizing the localizing and lateralizing yield of semiologic analysis of seizures, as has been previously attempted (21).

The SUDEP function cautions the encoder (and the reader of the report) of features known to increase the risk of SUDEP. Thus, it brings forth a prognostic issue of paramount importance that might be otherwise overlooked, highlighting yet another benefit of the automated report.

It is important to note that the semiologic extrapolation functions are designed to highlight key semiologic elements which may be of value in the diagnostic process. While most interpreters are epileptologists who should be aware of these features, the semiologic extrapolation function allows for reorganization of the sometimes multitude semiologic descriptors. In their current version, these functions are fraught with low sensitivity, which we hope to improve in the future by adding complex semiologic descriptors and incorporating EEG findings. We also wish to add simultaneous EEG encoding to DS, making it a more useful VEEG reporting tool, increasing its value in seizure annotation, and improving the semiologic extrapolation functions.

### Implementing DS in Machine Learning Algorithms

Automated video analysis is widely studied and has numerous applications, particularly given the vastly accumulating video data in our world. A popular approach to this problem is the implementation of deep learning algorithms. In these algorithms, rather than predefining features of interest, these features are learned from the data following training on annotated datasets. Currently available deep learning tools already achieve the main tasks for automatic video analysis, namely the detection and tracking of the keypoints (such as limbs, fingers, and facial landmarks) and temporal analysis of these keypoints with the goal to detect, recognize and characterize certain events of patient's action (22–25). Adapting these tools to tackle the problem of semiologic analysis of seizures would be crucial in achieving automated, or at least semiautomated, analysis of seizures, and in fact, some successful attempts have already been made (26). In turn, automatic analysis of seizure semiology will aid in making VEM more widely available and is particularly applicable in the context of home VEM. Here we have demonstrated that the DS software can aid in the structured analysis of video excerpts with the potential of forming an annotated epilepsy-oriented video database that can be used by deep learning algorithms.

### Digital Semiology as an Open-Source Software and Community

Open-source initiatives are gaining popularity in neuroscience (27, 28). By publishing the DS software as open-source code, we hope to kindle a community of users working together to improve the software, making standardized semiologic reporting easy and accessible. This may aid in achieving two goals: standardizing semiologic assessment and amassing annotated video episodes. Making DS an open project ensures that the gained advances are available to the scientific community, facilitating the distribution of knowledge.

The DS software also highlights the potential of automated and semiautomated analysis in diagnosis and prognosis, as seen in the semiologic extrapolation functions. The open-source approach will enable the development and implementation of new such features. Through a joint effort of users, DS may evolve into a powerful clinical and research tool, combining standardized semiologic encoding and clinical alerts.

This study has several limitations. Since it is a pilot study and only 60 events were encoded using the software, it may not encompass the full breadth of semiologic descriptions. In addition, to overcome the issue of inter-rater variability in semiologic encoding, the episodes encoded in the study had previously been seen by the interpreting epileptologist, alongside the EEG. Although at least 6 months had elapsed between the encoding of the two reports, it cannot be ruled out that the encoding physician had some recollection of the episodes. The addition of concurrent EEG findings which are now lacking, will improve report precision, allow implementation of the 2017 ILAE classification scheme in the software, and improve the sensitivity of the semiologic extrapolation functions.

To conclude, in this innovative pilot study, we have shown that the DS software can be successfully implemented to create reports for video excerpts in an adult and pediatric epilepsy cohort. The structured report is ideal for identification of specific semiologic characteristics and sequences, as shown in the semiologic extrapolation function. Finally, formation of an annotated epilepsy video database using DS can be used to further the goal of automating VEM analysis.

## Data Availability Statement

The raw data supporting the conclusions of this article will be made available by the authors, without undue reservation.

## Ethics Statement

The studies involving human participants were reviewed and approved by Institutional Helsinki Committee, Hadassah Medical Center. Written informed consent from the participants' legal guardian/next of kin was not required to participate in this study in accordance with the national legislation and the institutional requirements.

## Author Contributions

TB contributed to specifying the software, encoded episodes, performed data analysis, and wrote the initial version of the manuscript. TG encoded episodes and critically reviewed the manuscript. PH-S programmed the software. RZ and BK critically reviewed the manuscript. ET contributed to the writing of the manuscript. OA contributed to programming and data analysis and critically reviewed the manuscript. DE conceptualized the software, encoded episodes, and contributed to the writing of the manuscript. MM conceptualized and programmed the software, encoded episodes, and contributed to the writing of the manuscript. All authors contributed to the article and approved the submitted version.

## Funding

The research was supported by the Prusiner-Abramsky research award and the Orion foundation.

## Conflict of Interest

The authors declare that the research was conducted in the absence of any commercial or financial relationships that could be construed as a potential conflict of interest.

## Publisher's Note

All claims expressed in this article are solely those of the authors and do not necessarily represent those of their affiliated organizations, or those of the publisher, the editors and the reviewers. Any product that may be evaluated in this article, or claim that may be made by its manufacturer, is not guaranteed or endorsed by the publisher.
