# Supplementary material for: Digital Semiology: A Prototype for Standardized, Computer-Based Semiologic Encoding of Seizures
Source: Front Neurol. 2021 Oct 5;12:711378. doi: 10.3389/fneur.2021.711378 (PMC8525609; doi:10.3389/fneur.2021.711378)
Supplement: Supplementary file 1 [file Presentation_1.PPTX]

## Slide 1
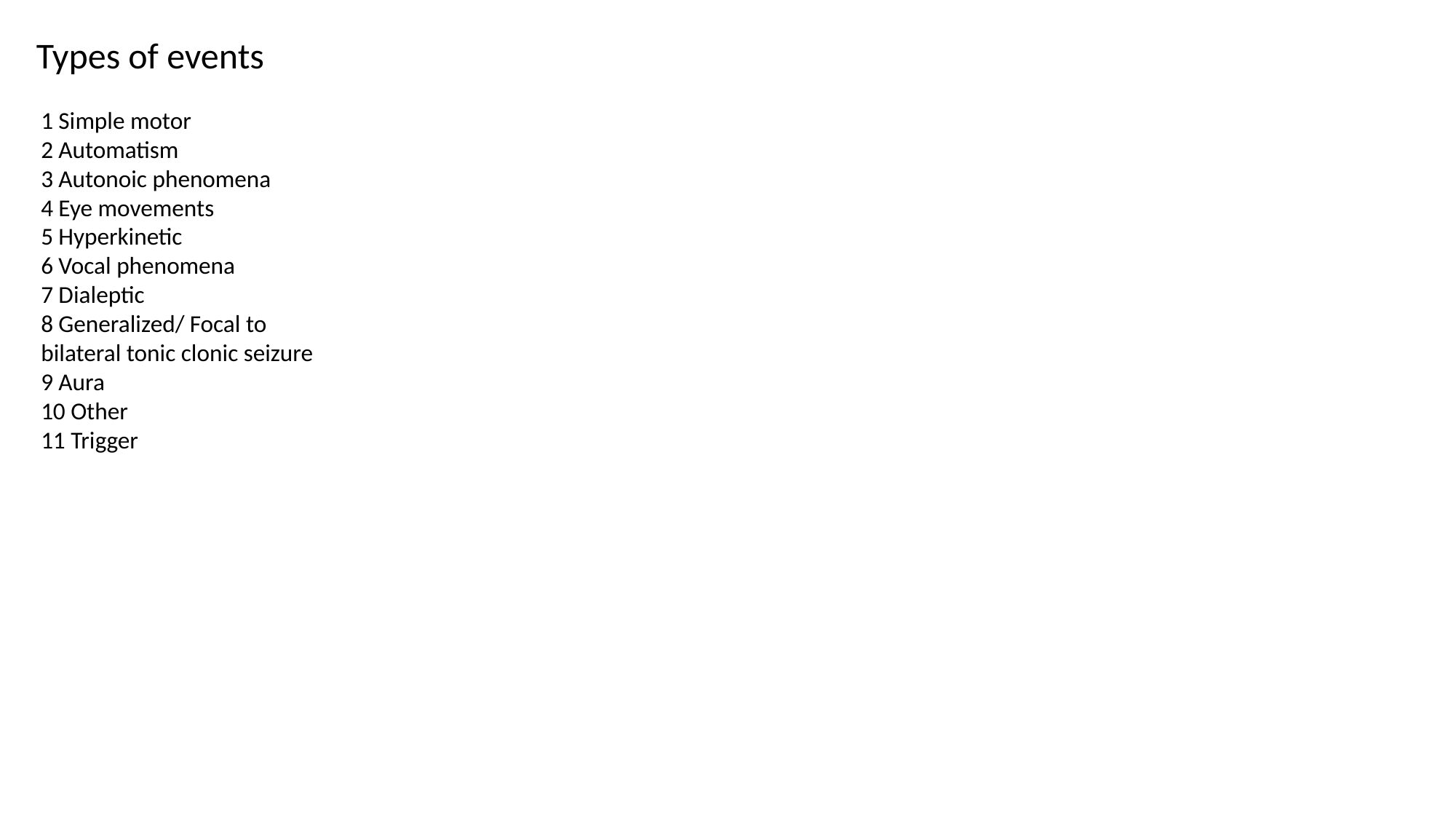

Types of events
1 Simple motor
2 Automatism
3 Autonoic phenomena
4 Eye movements
5 Hyperkinetic
6 Vocal phenomena
7 Dialeptic
8 Generalized/ Focal to bilateral tonic clonic seizure
9 Aura
10 Other
11 Trigger

## Slide 2
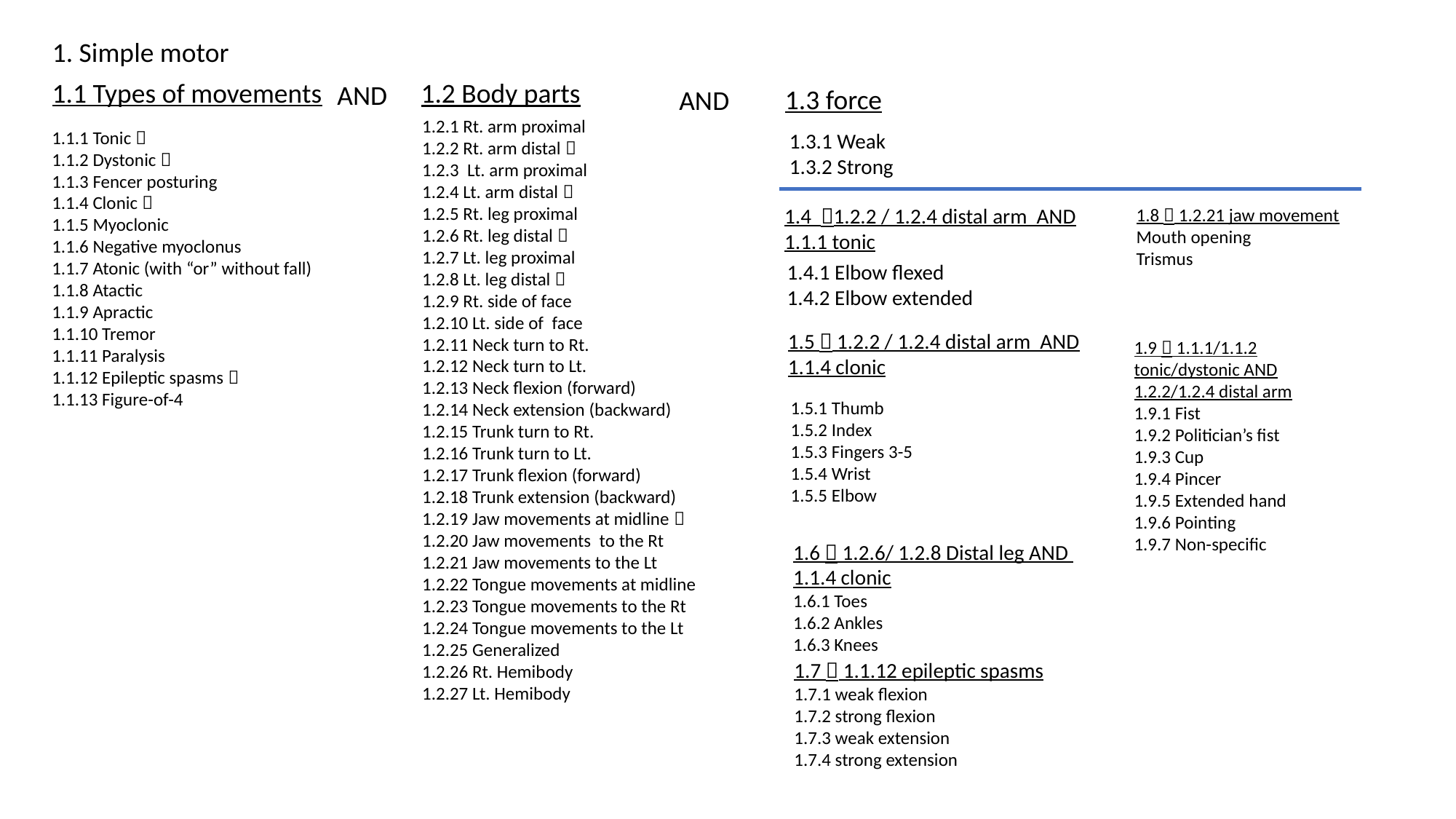

1. Simple motor
1.1 Types of movements
1.2 Body parts
AND
1.3 force
AND
1.2.1 Rt. arm proximal
1.2.2 Rt. arm distal 
1.2.3 Lt. arm proximal
1.2.4 Lt. arm distal 
1.2.5 Rt. leg proximal
1.2.6 Rt. leg distal 
1.2.7 Lt. leg proximal
1.2.8 Lt. leg distal 
1.2.9 Rt. side of face
1.2.10 Lt. side of face
1.2.11 Neck turn to Rt.
1.2.12 Neck turn to Lt.
1.2.13 Neck flexion (forward)
1.2.14 Neck extension (backward)
1.2.15 Trunk turn to Rt.
1.2.16 Trunk turn to Lt.
1.2.17 Trunk flexion (forward)
1.2.18 Trunk extension (backward)
1.2.19 Jaw movements at midline 
1.2.20 Jaw movements to the Rt
1.2.21 Jaw movements to the Lt
1.2.22 Tongue movements at midline
1.2.23 Tongue movements to the Rt
1.2.24 Tongue movements to the Lt
1.2.25 Generalized
1.2.26 Rt. Hemibody
1.2.27 Lt. Hemibody
1.1.1 Tonic 
1.1.2 Dystonic 
1.1.3 Fencer posturing
1.1.4 Clonic 
1.1.5 Myoclonic
1.1.6 Negative myoclonus
1.1.7 Atonic (with “or” without fall)
1.1.8 Atactic
1.1.9 Apractic
1.1.10 Tremor
1.1.11 Paralysis
1.1.12 Epileptic spasms 
1.1.13 Figure-of-4
1.3.1 Weak
1.3.2 Strong
1.4 1.2.2 / 1.2.4 distal arm AND 1.1.1 tonic
1.8  1.2.21 jaw movement
Mouth opening
Trismus
1.4.1 Elbow flexed
1.4.2 Elbow extended
1.5  1.2.2 / 1.2.4 distal arm AND 1.1.4 clonic
1.9  1.1.1/1.1.2 tonic/dystonic AND 1.2.2/1.2.4 distal arm
1.9.1 Fist
1.9.2 Politician’s fist
1.9.3 Cup
1.9.4 Pincer
1.9.5 Extended hand
1.9.6 Pointing
1.9.7 Non-specific
1.5.1 Thumb
1.5.2 Index
1.5.3 Fingers 3-5
1.5.4 Wrist
1.5.5 Elbow
1.6  1.2.6/ 1.2.8 Distal leg AND
1.1.4 clonic
1.6.1 Toes
1.6.2 Ankles
1.6.3 Knees
1.7  1.1.12 epileptic spasms
1.7.1 weak flexion
1.7.2 strong flexion
1.7.3 weak extension
1.7.4 strong extension

## Slide 3
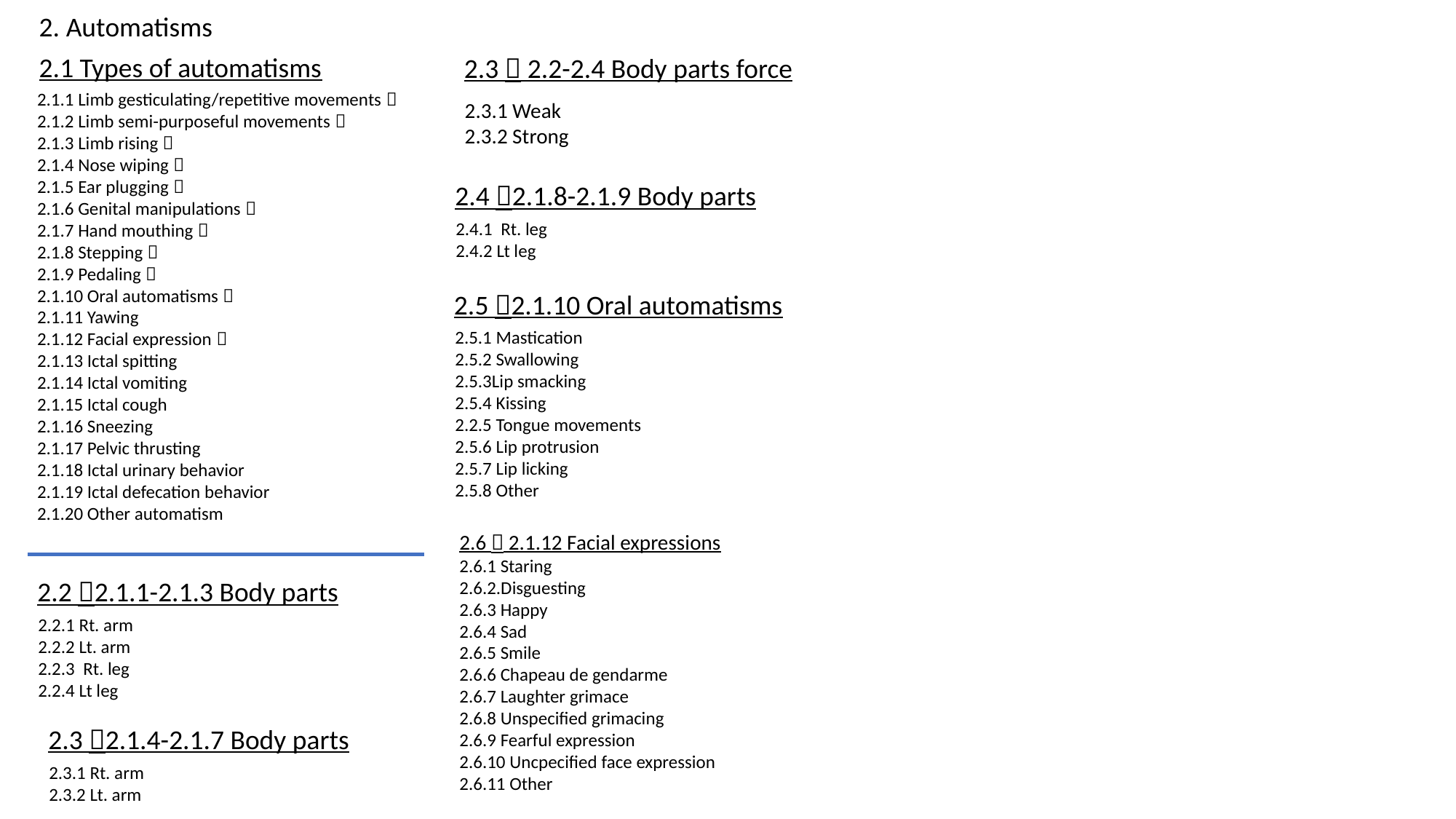

2. Automatisms
2.1 Types of automatisms
2.3  2.2-2.4 Body parts force
2.1.1 Limb gesticulating/repetitive movements 
2.1.2 Limb semi-purposeful movements 
2.1.3 Limb rising 
2.1.4 Nose wiping 
2.1.5 Ear plugging 
2.1.6 Genital manipulations 
2.1.7 Hand mouthing 
2.1.8 Stepping 
2.1.9 Pedaling 
2.1.10 Oral automatisms 
2.1.11 Yawing
2.1.12 Facial expression 
2.1.13 Ictal spitting
2.1.14 Ictal vomiting
2.1.15 Ictal cough
2.1.16 Sneezing
2.1.17 Pelvic thrusting
2.1.18 Ictal urinary behavior
2.1.19 Ictal defecation behavior
2.1.20 Other automatism
2.3.1 Weak
2.3.2 Strong
2.4 2.1.8-2.1.9 Body parts
2.4.1 Rt. leg
2.4.2 Lt leg
2.5 2.1.10 Oral automatisms
2.5.1 Mastication
2.5.2 Swallowing
2.5.3Lip smacking
2.5.4 Kissing
2.2.5 Tongue movements
2.5.6 Lip protrusion
2.5.7 Lip licking
2.5.8 Other
2.6  2.1.12 Facial expressions
2.6.1 Staring
2.6.2.Disguesting
2.6.3 Happy
2.6.4 Sad
2.6.5 Smile
2.6.6 Chapeau de gendarme
2.6.7 Laughter grimace
2.6.8 Unspecified grimacing
2.6.9 Fearful expression
2.6.10 Uncpecified face expression
2.6.11 Other
2.2 2.1.1-2.1.3 Body parts
2.2.1 Rt. arm
2.2.2 Lt. arm
2.2.3 Rt. leg
2.2.4 Lt leg
2.3 2.1.4-2.1.7 Body parts
2.3.1 Rt. arm
2.3.2 Lt. arm

## Slide 4
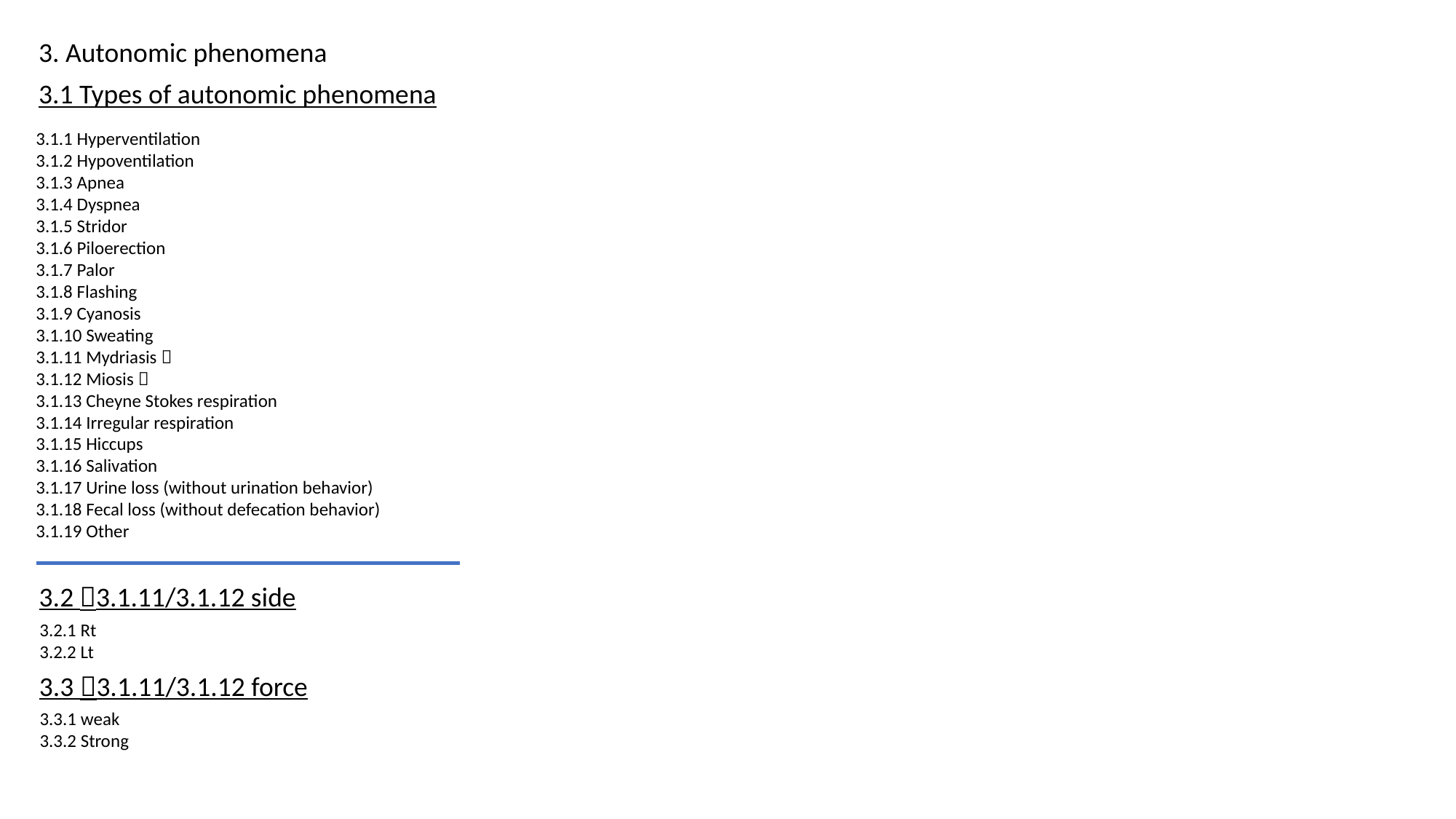

3. Autonomic phenomena
3.1 Types of autonomic phenomena
3.1.1 Hyperventilation
3.1.2 Hypoventilation
3.1.3 Apnea
3.1.4 Dyspnea
3.1.5 Stridor
3.1.6 Piloerection
3.1.7 Palor
3.1.8 Flashing
3.1.9 Cyanosis
3.1.10 Sweating
3.1.11 Mydriasis 
3.1.12 Miosis 
3.1.13 Cheyne Stokes respiration
3.1.14 Irregular respiration
3.1.15 Hiccups
3.1.16 Salivation
3.1.17 Urine loss (without urination behavior)
3.1.18 Fecal loss (without defecation behavior)
3.1.19 Other
3.2 3.1.11/3.1.12 side
3.2.1 Rt
3.2.2 Lt
3.3 3.1.11/3.1.12 force
3.3.1 weak
3.3.2 Strong

## Slide 5
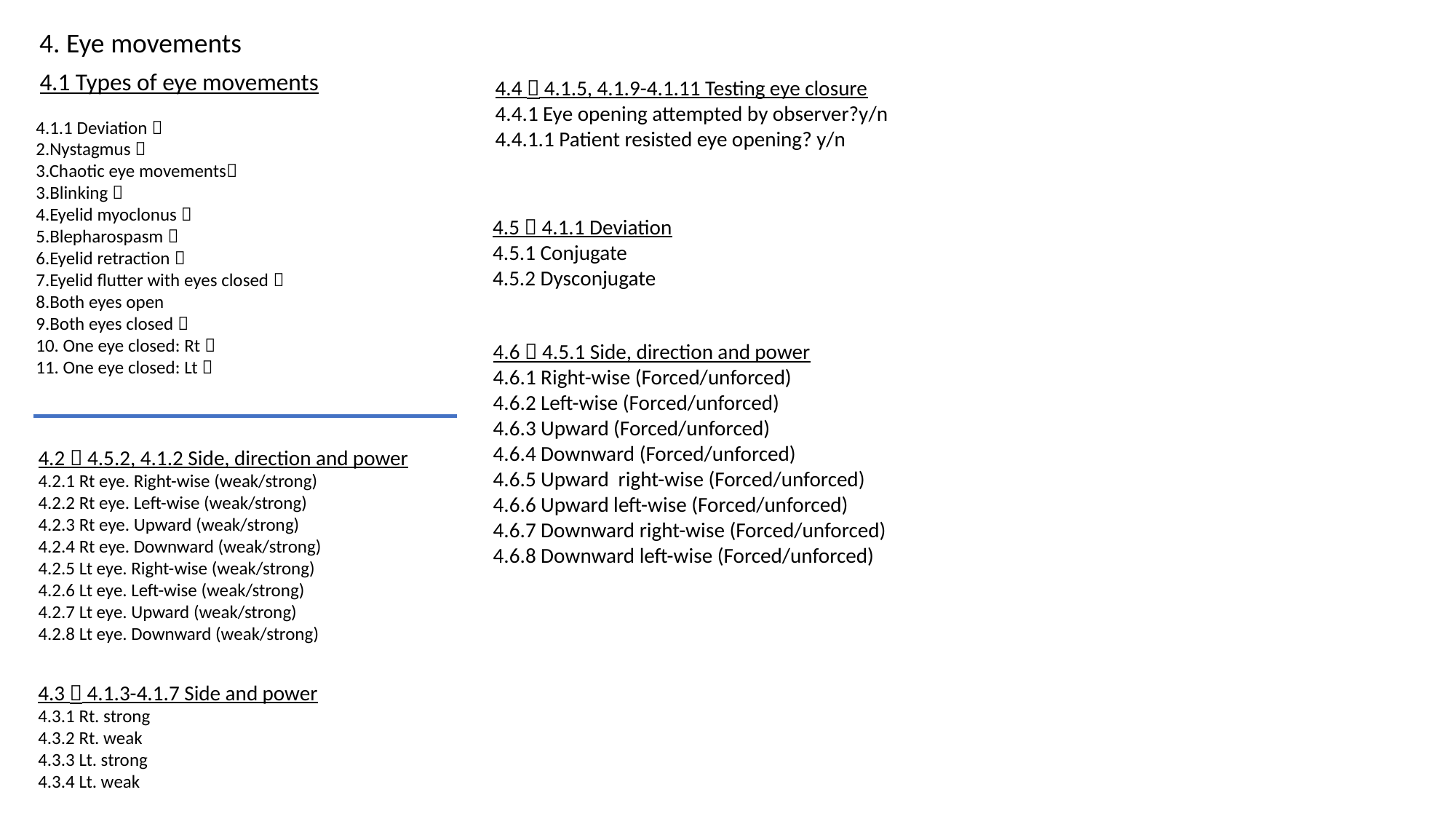

4. Eye movements
4.1 Types of eye movements
4.4  4.1.5, 4.1.9-4.1.11 Testing eye closure
4.4.1 Eye opening attempted by observer?y/n
4.4.1.1 Patient resisted eye opening? y/n
4.1.1 Deviation 
2.Nystagmus 
3.Chaotic eye movements
3.Blinking 
4.Eyelid myoclonus 
5.Blepharospasm 
6.Eyelid retraction 
7.Eyelid flutter with eyes closed 
8.Both eyes open
9.Both eyes closed 
10. One eye closed: Rt 
11. One eye closed: Lt 
4.5  4.1.1 Deviation
4.5.1 Conjugate
4.5.2 Dysconjugate
4.6  4.5.1 Side, direction and power
4.6.1 Right-wise (Forced/unforced)
4.6.2 Left-wise (Forced/unforced)
4.6.3 Upward (Forced/unforced)
4.6.4 Downward (Forced/unforced)
4.6.5 Upward right-wise (Forced/unforced)
4.6.6 Upward left-wise (Forced/unforced)
4.6.7 Downward right-wise (Forced/unforced)
4.6.8 Downward left-wise (Forced/unforced)
4.2  4.5.2, 4.1.2 Side, direction and power
4.2.1 Rt eye. Right-wise (weak/strong)
4.2.2 Rt eye. Left-wise (weak/strong)
4.2.3 Rt eye. Upward (weak/strong)
4.2.4 Rt eye. Downward (weak/strong)
4.2.5 Lt eye. Right-wise (weak/strong)
4.2.6 Lt eye. Left-wise (weak/strong)
4.2.7 Lt eye. Upward (weak/strong)
4.2.8 Lt eye. Downward (weak/strong)
4.3  4.1.3-4.1.7 Side and power
4.3.1 Rt. strong
4.3.2 Rt. weak
4.3.3 Lt. strong
4.3.4 Lt. weak

## Slide 6
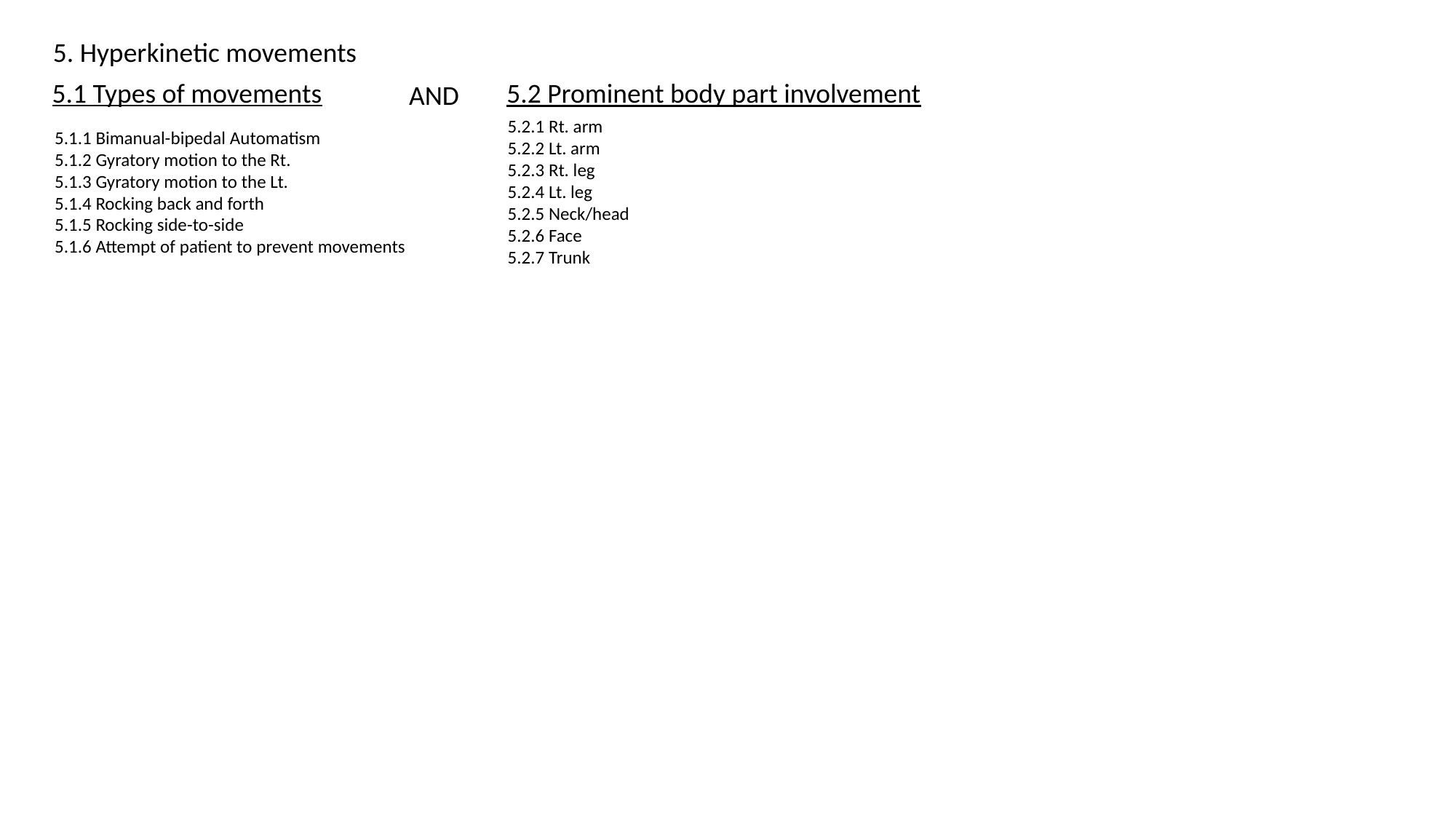

5. Hyperkinetic movements
5.1 Types of movements
5.2 Prominent body part involvement
AND
5.2.1 Rt. arm
5.2.2 Lt. arm
5.2.3 Rt. leg
5.2.4 Lt. leg
5.2.5 Neck/head
5.2.6 Face
5.2.7 Trunk
5.1.1 Bimanual-bipedal Automatism
5.1.2 Gyratory motion to the Rt.
5.1.3 Gyratory motion to the Lt.
5.1.4 Rocking back and forth
5.1.5 Rocking side-to-side
5.1.6 Attempt of patient to prevent movements

## Slide 7
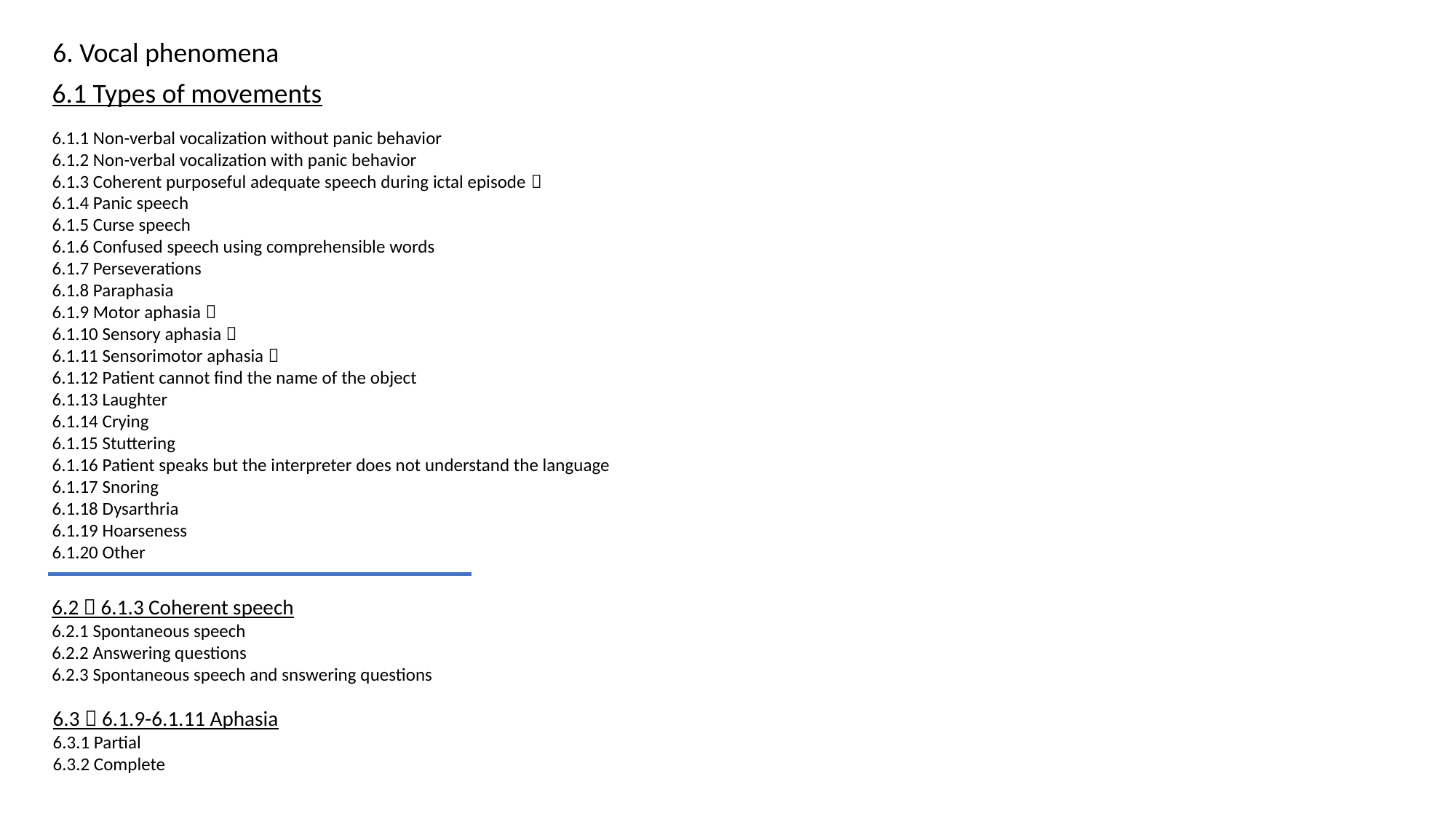

6. Vocal phenomena
6.1 Types of movements
6.1.1 Non-verbal vocalization without panic behavior
6.1.2 Non-verbal vocalization with panic behavior
6.1.3 Coherent purposeful adequate speech during ictal episode 
6.1.4 Panic speech
6.1.5 Curse speech
6.1.6 Confused speech using comprehensible words
6.1.7 Perseverations
6.1.8 Paraphasia
6.1.9 Motor aphasia 
6.1.10 Sensory aphasia 
6.1.11 Sensorimotor aphasia 
6.1.12 Patient cannot find the name of the object
6.1.13 Laughter
6.1.14 Crying
6.1.15 Stuttering
6.1.16 Patient speaks but the interpreter does not understand the language
6.1.17 Snoring
6.1.18 Dysarthria
6.1.19 Hoarseness
6.1.20 Other
6.2  6.1.3 Coherent speech
6.2.1 Spontaneous speech
6.2.2 Answering questions
6.2.3 Spontaneous speech and snswering questions
6.3  6.1.9-6.1.11 Aphasia
6.3.1 Partial
6.3.2 Complete

## Slide 8
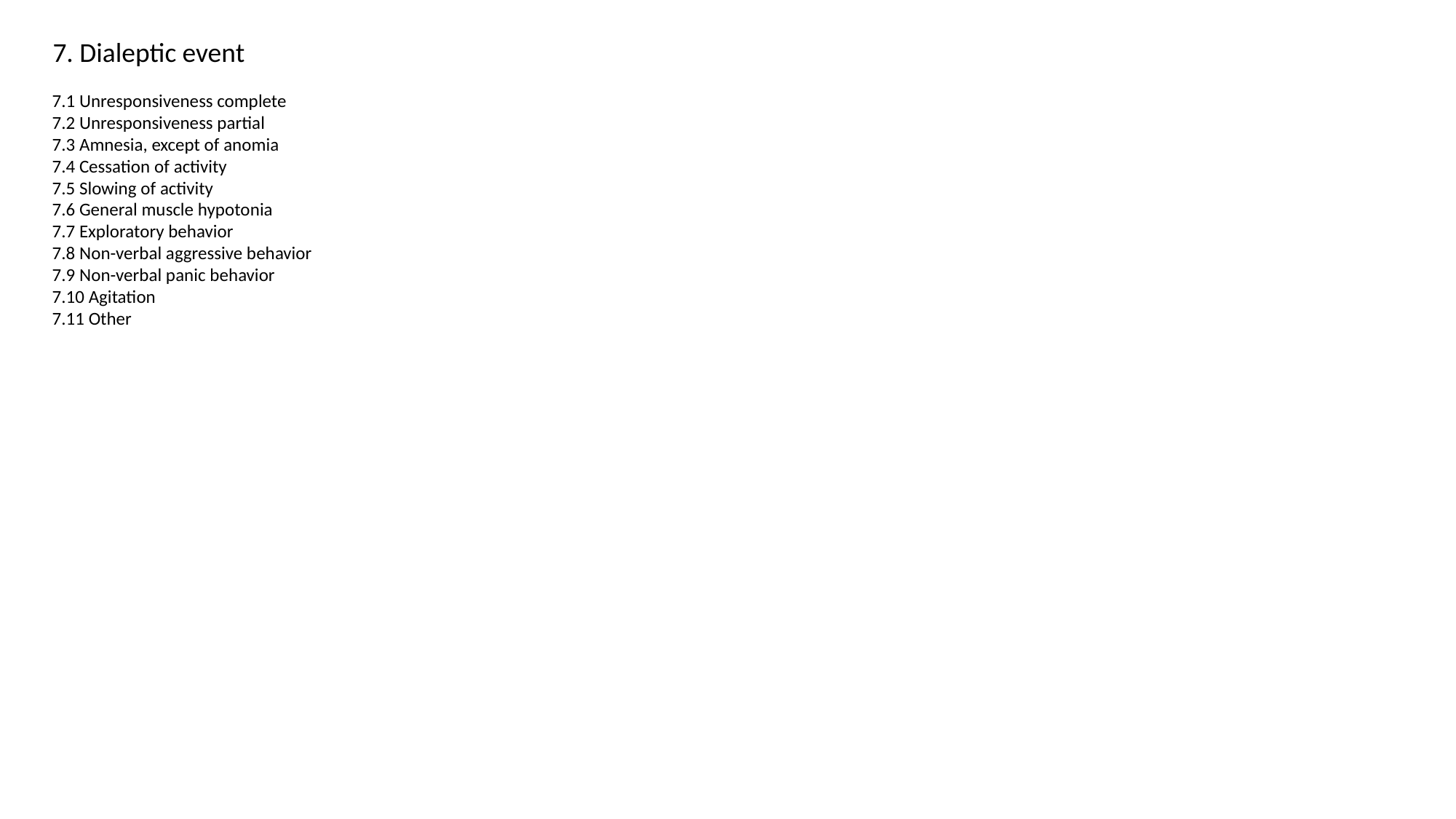

7. Dialeptic event
7.1 Unresponsiveness complete
7.2 Unresponsiveness partial
7.3 Amnesia, except of anomia
7.4 Cessation of activity
7.5 Slowing of activity
7.6 General muscle hypotonia
7.7 Exploratory behavior
7.8 Non-verbal aggressive behavior
7.9 Non-verbal panic behavior
7.10 Agitation
7.11 Other

## Slide 9
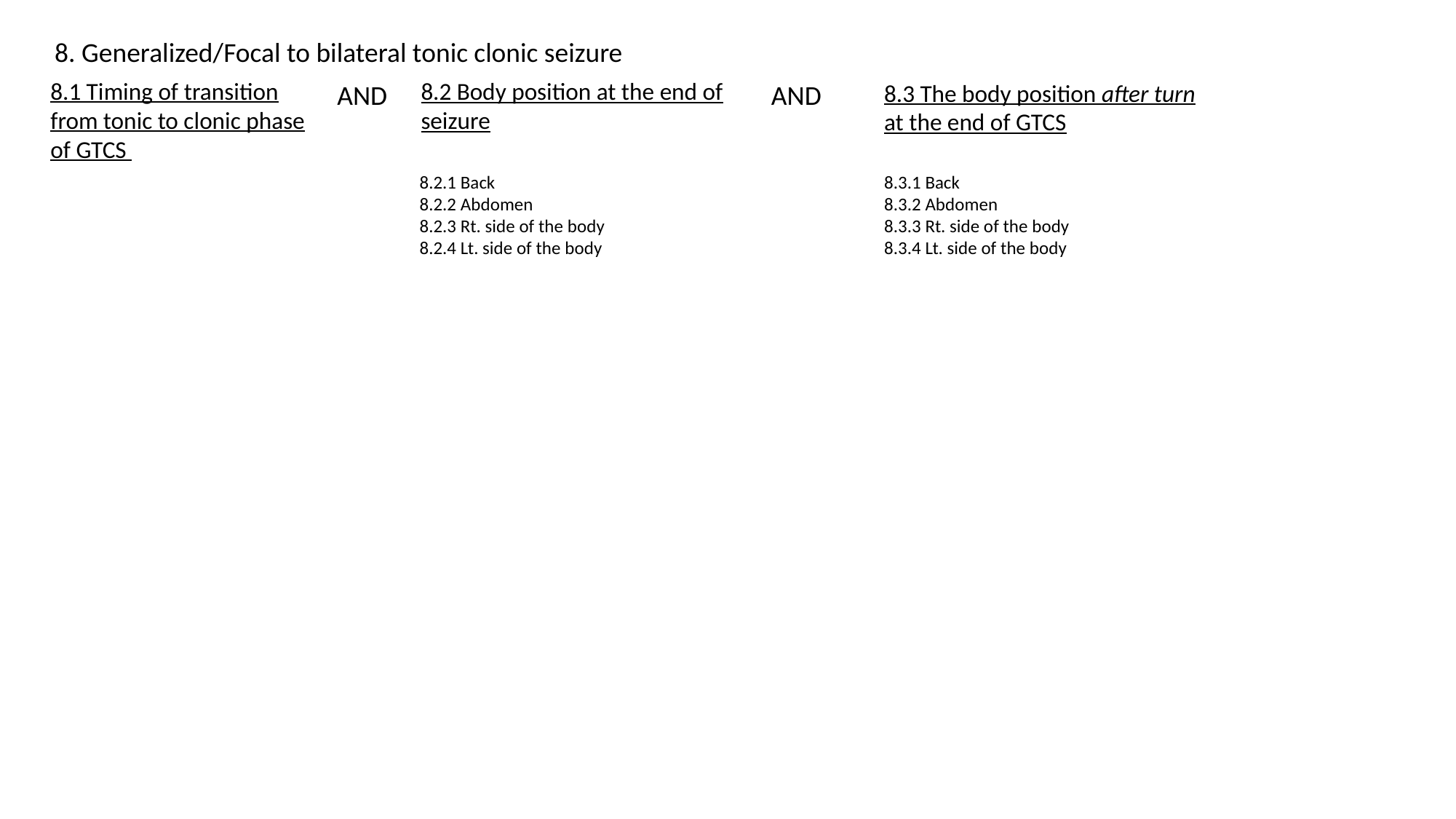

8. Generalized/Focal to bilateral tonic clonic seizure
8.1 Timing of transition from tonic to clonic phase of GTCS
8.2 Body position at the end of seizure
AND
AND
8.3 The body position after turn at the end of GTCS
8.2.1 Back
8.2.2 Abdomen
8.2.3 Rt. side of the body
8.2.4 Lt. side of the body
8.3.1 Back
8.3.2 Abdomen
8.3.3 Rt. side of the body
8.3.4 Lt. side of the body

## Slide 10
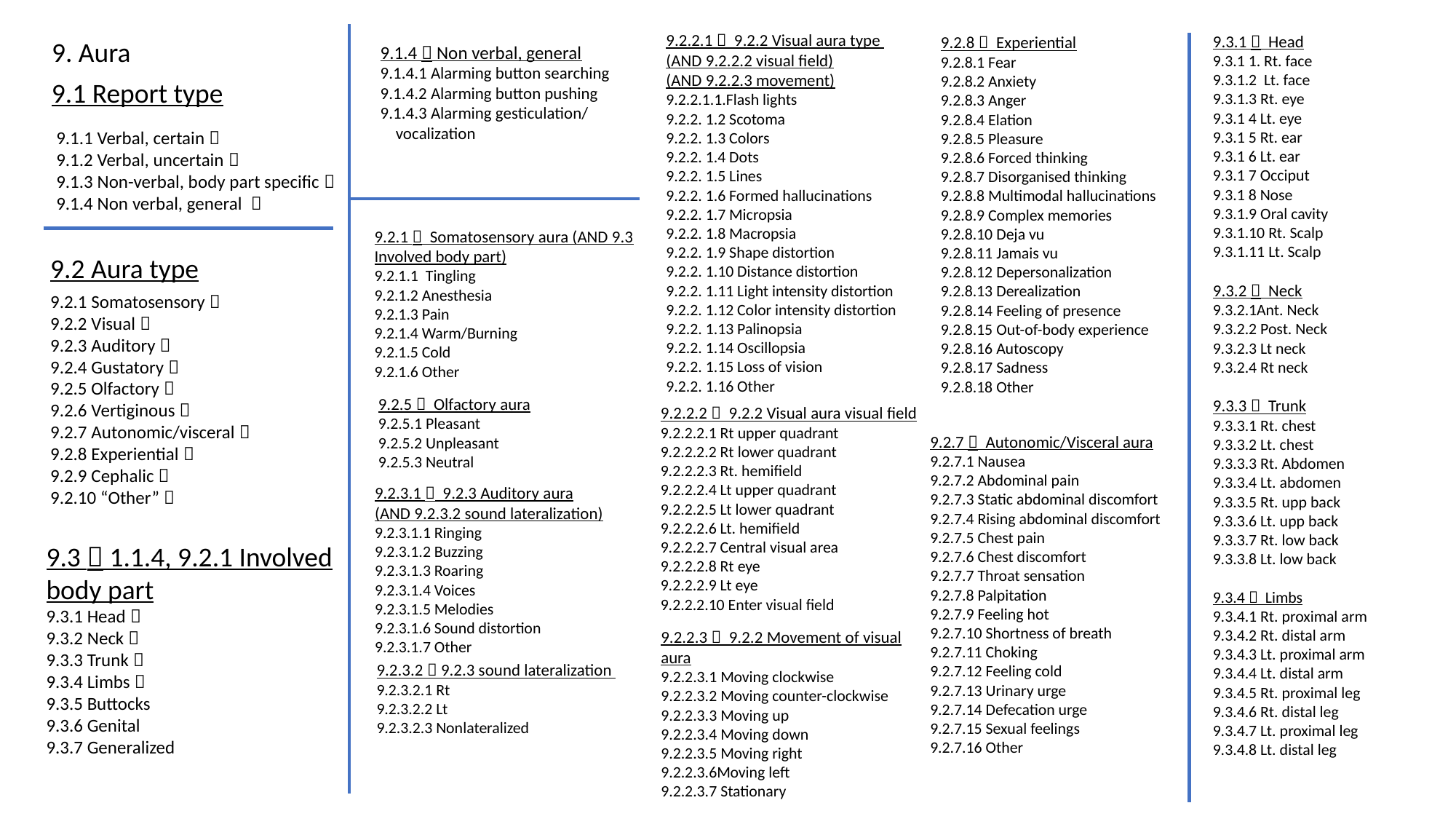

9.2.2.1  9.2.2 Visual aura type
(AND 9.2.2.2 visual field)
(AND 9.2.2.3 movement)
9.2.2.1.1.Flash lights
9.2.2. 1.2 Scotoma
9.2.2. 1.3 Colors
9.2.2. 1.4 Dots
9.2.2. 1.5 Lines
9.2.2. 1.6 Formed hallucinations
9.2.2. 1.7 Micropsia
9.2.2. 1.8 Macropsia
9.2.2. 1.9 Shape distortion
9.2.2. 1.10 Distance distortion
9.2.2. 1.11 Light intensity distortion
9.2.2. 1.12 Color intensity distortion
9.2.2. 1.13 Palinopsia
9.2.2. 1.14 Oscillopsia
9.2.2. 1.15 Loss of vision
9.2.2. 1.16 Other
9.3.1  Head
9.3.1 1. Rt. face
9.3.1.2 Lt. face
9.3.1.3 Rt. eye
9.3.1 4 Lt. eye
9.3.1 5 Rt. ear
9.3.1 6 Lt. ear
9.3.1 7 Occiput
9.3.1 8 Nose
9.3.1.9 Oral cavity
9.3.1.10 Rt. Scalp
9.3.1.11 Lt. Scalp
9.3.2  Neck
9.3.2.1Ant. Neck
9.3.2.2 Post. Neck
9.3.2.3 Lt neck
9.3.2.4 Rt neck
9.3.3  Trunk
9.3.3.1 Rt. chest
9.3.3.2 Lt. chest
9.3.3.3 Rt. Abdomen
9.3.3.4 Lt. abdomen
9.3.3.5 Rt. upp back
9.3.3.6 Lt. upp back
9.3.3.7 Rt. low back
9.3.3.8 Lt. low back
9.3.4  Limbs
9.3.4.1 Rt. proximal arm
9.3.4.2 Rt. distal arm
9.3.4.3 Lt. proximal arm
9.3.4.4 Lt. distal arm
9.3.4.5 Rt. proximal leg
9.3.4.6 Rt. distal leg
9.3.4.7 Lt. proximal leg
9.3.4.8 Lt. distal leg
9.2.8  Experiential
9.2.8.1 Fear
9.2.8.2 Anxiety
9.2.8.3 Anger
9.2.8.4 Elation
9.2.8.5 Pleasure
9.2.8.6 Forced thinking
9.2.8.7 Disorganised thinking
9.2.8.8 Multimodal hallucinations
9.2.8.9 Complex memories
9.2.8.10 Deja vu
9.2.8.11 Jamais vu
9.2.8.12 Depersonalization
9.2.8.13 Derealization
9.2.8.14 Feeling of presence
9.2.8.15 Out-of-body experience
9.2.8.16 Autoscopy
9.2.8.17 Sadness
9.2.8.18 Other
9. Aura
9.1.4  Non verbal, general
9.1.4.1 Alarming button searching
9.1.4.2 Alarming button pushing
9.1.4.3 Alarming gesticulation/
 vocalization
9.1 Report type
9.1.1 Verbal, certain 
9.1.2 Verbal, uncertain 
9.1.3 Non-verbal, body part specific 
9.1.4 Non verbal, general 
9.2.1  Somatosensory aura (AND 9.3 Involved body part)
9.2.1.1 Tingling
9.2.1.2 Anesthesia
9.2.1.3 Pain
9.2.1.4 Warm/Burning
9.2.1.5 Cold
9.2.1.6 Other
9.2 Aura type
9.2.1 Somatosensory 
9.2.2 Visual 
9.2.3 Auditory 
9.2.4 Gustatory 
9.2.5 Olfactory 
9.2.6 Vertiginous 
9.2.7 Autonomic/visceral 
9.2.8 Experiential 
9.2.9 Cephalic 
9.2.10 “Other” 
9.2.5  Olfactory aura
9.2.5.1 Pleasant
9.2.5.2 Unpleasant
9.2.5.3 Neutral
9.2.2.2  9.2.2 Visual aura visual field
9.2.2.2.1 Rt upper quadrant
9.2.2.2.2 Rt lower quadrant
9.2.2.2.3 Rt. hemifield
9.2.2.2.4 Lt upper quadrant
9.2.2.2.5 Lt lower quadrant
9.2.2.2.6 Lt. hemifield
9.2.2.2.7 Central visual area
9.2.2.2.8 Rt eye
9.2.2.2.9 Lt eye
9.2.2.2.10 Enter visual field
9.2.7  Autonomic/Visceral aura
9.2.7.1 Nausea
9.2.7.2 Abdominal pain
9.2.7.3 Static abdominal discomfort
9.2.7.4 Rising abdominal discomfort
9.2.7.5 Chest pain
9.2.7.6 Chest discomfort
9.2.7.7 Throat sensation
9.2.7.8 Palpitation
9.2.7.9 Feeling hot
9.2.7.10 Shortness of breath
9.2.7.11 Choking
9.2.7.12 Feeling cold
9.2.7.13 Urinary urge
9.2.7.14 Defecation urge
9.2.7.15 Sexual feelings
9.2.7.16 Other
9.2.3.1  9.2.3 Auditory aura
(AND 9.2.3.2 sound lateralization)
9.2.3.1.1 Ringing
9.2.3.1.2 Buzzing
9.2.3.1.3 Roaring
9.2.3.1.4 Voices
9.2.3.1.5 Melodies
9.2.3.1.6 Sound distortion
9.2.3.1.7 Other
9.3  1.1.4, 9.2.1 Involved body part
9.3.1 Head 
9.3.2 Neck 
9.3.3 Trunk 
9.3.4 Limbs 
9.3.5 Buttocks
9.3.6 Genital
9.3.7 Generalized
9.2.2.3  9.2.2 Movement of visual aura
9.2.2.3.1 Moving clockwise
9.2.2.3.2 Moving counter-clockwise
9.2.2.3.3 Moving up
9.2.2.3.4 Moving down
9.2.2.3.5 Moving right
9.2.2.3.6Moving left
9.2.2.3.7 Stationary
9.2.3.2  9.2.3 sound lateralization
9.2.3.2.1 Rt
9.2.3.2.2 Lt
9.2.3.2.3 Nonlateralized

## Slide 11
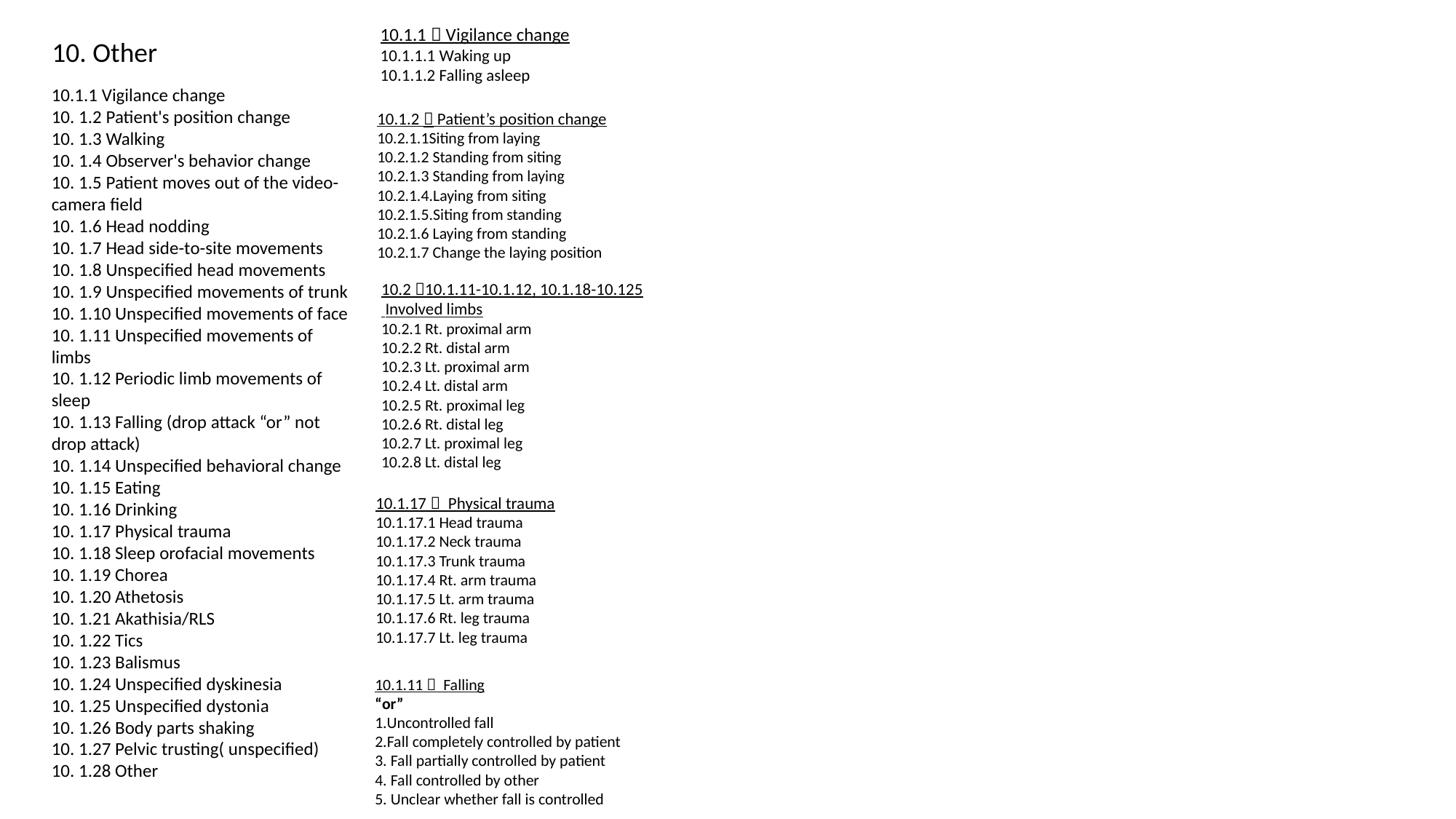

10.1.1  Vigilance change
10.1.1.1 Waking up
10.1.1.2 Falling asleep
10. Other
10.1.1 Vigilance change
10. 1.2 Patient's position change
10. 1.3 Walking
10. 1.4 Observer's behavior change
10. 1.5 Patient moves out of the video-camera field
10. 1.6 Head nodding
10. 1.7 Head side-to-site movements
10. 1.8 Unspecified head movements
10. 1.9 Unspecified movements of trunk
10. 1.10 Unspecified movements of face
10. 1.11 Unspecified movements of limbs
10. 1.12 Periodic limb movements of sleep
10. 1.13 Falling (drop attack “or” not drop attack)
10. 1.14 Unspecified behavioral change
10. 1.15 Eating
10. 1.16 Drinking
10. 1.17 Physical trauma
10. 1.18 Sleep orofacial movements
10. 1.19 Chorea
10. 1.20 Athetosis
10. 1.21 Akathisia/RLS
10. 1.22 Tics
10. 1.23 Balismus
10. 1.24 Unspecified dyskinesia
10. 1.25 Unspecified dystonia
10. 1.26 Body parts shaking
10. 1.27 Pelvic trusting( unspecified)
10. 1.28 Other
10.1.2  Patient’s position change
10.2.1.1Siting from laying
10.2.1.2 Standing from siting
10.2.1.3 Standing from laying
10.2.1.4.Laying from siting
10.2.1.5.Siting from standing
10.2.1.6 Laying from standing
10.2.1.7 Change the laying position
10.2 10.1.11-10.1.12, 10.1.18-10.125
 Involved limbs
10.2.1 Rt. proximal arm
10.2.2 Rt. distal arm
10.2.3 Lt. proximal arm
10.2.4 Lt. distal arm
10.2.5 Rt. proximal leg
10.2.6 Rt. distal leg
10.2.7 Lt. proximal leg
10.2.8 Lt. distal leg
10.1.17  Physical trauma
10.1.17.1 Head trauma
10.1.17.2 Neck trauma
10.1.17.3 Trunk trauma
10.1.17.4 Rt. arm trauma
10.1.17.5 Lt. arm trauma
10.1.17.6 Rt. leg trauma
10.1.17.7 Lt. leg trauma
10.1.11  Falling
“or”
1.Uncontrolled fall
2.Fall completely controlled by patient
3. Fall partially controlled by patient
4. Fall controlled by other
5. Unclear whether fall is controlled

## Slide 12
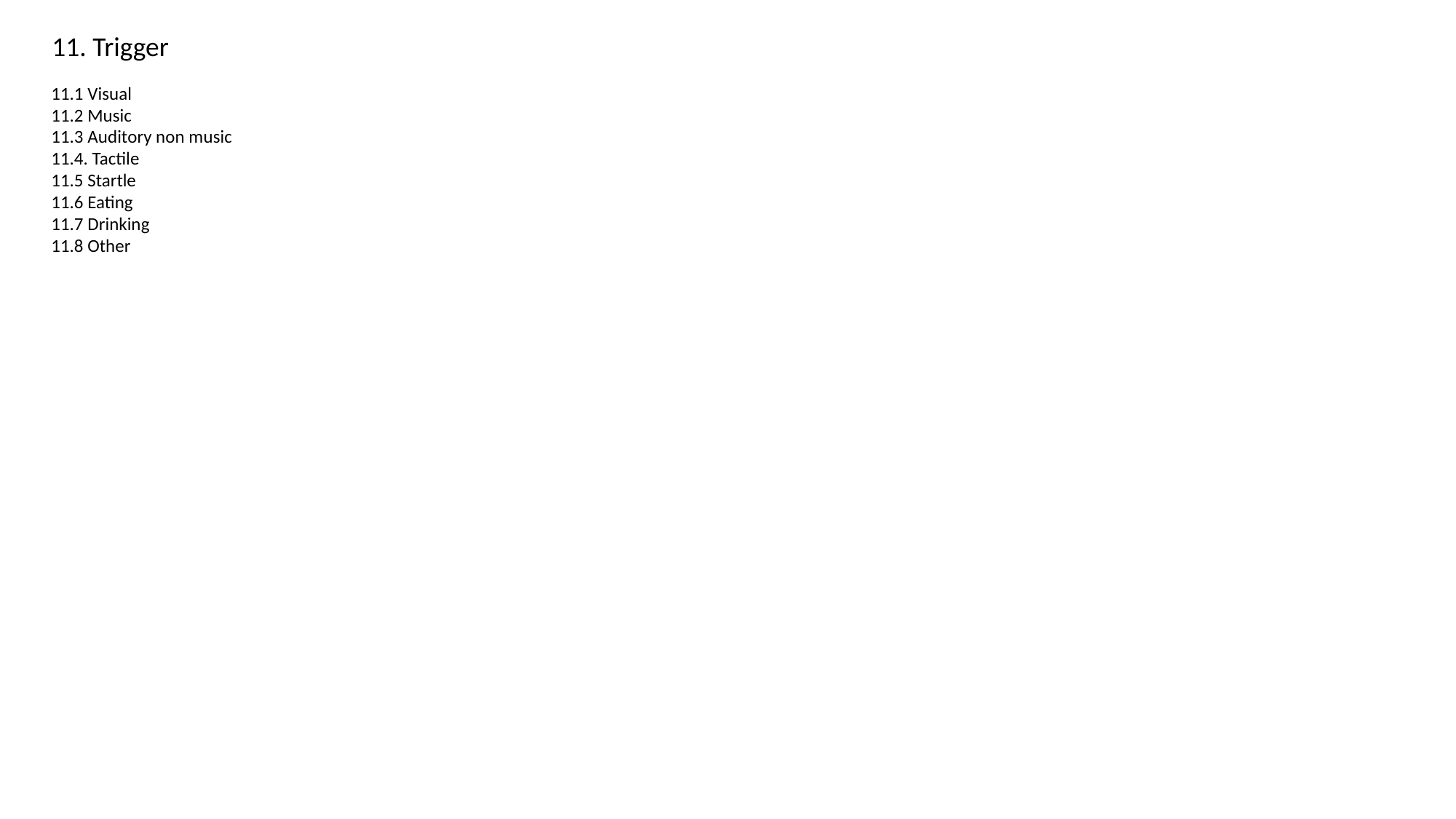

11. Trigger
11.1 Visual
11.2 Music
11.3 Auditory non music
11.4. Tactile
11.5 Startle
11.6 Eating
11.7 Drinking
11.8 Other
